# Toxicity in combined therapies for tumours treatments: a lesson from BAG3 in the TME?

**DOI:** 10.3389/fimmu.2023.1241543

**Published:** 2023-07-20

**Authors:** Alessandra Rosati, Liberato Marzullo, Margot De Marco, Vincenzo De Laurenzi, Maria Francesca D’Amico, Maria Caterina Turco

**Affiliations:** ^1^Department of Medicine, Surgery and Dentistry “Schola Medica Salernitana”, University of Salerno, Baronissi, SA, Italy; ^2^Department of Innovative Technologies in Medicine and Dentistry, “G. d’Annunzio” University of Chieti-Pescara, Chieti, Italy; ^3^Azienda Ospedaliera di Rilievo Nazionale e di Alta Specialità San Giuseppe Moscati (A.O.S.G.), Avellino, Italy

**Keywords:** TME, tumours, combined therapies, toxicity, BAG3

## Introduction

In tumours treatment, targeted therapies offer the opportunity to design therapeutic combinations by exploiting the different mechanisms of action of the drugs used. However, an increase in toxicity is also often observed alongside the therapeutic synergisms. For example, combination of immune checkpoint inhibitors (ICIs) with angiogenesis inhibitors (AGIs) shows synergistic antitumour effects, but also a higher risk of cardiovascular adverse events than treatments with ICIs alone (1). As well, combining ICIs with DNA damage repair inhibitors (DDRIs) in the treatment of urologic cancers greatly increases the risk of toxicity and side effects (2). In pediatric patients affected by primary CNS or PNS tumours, MEK inhibitor (MEKI) (trametinib)- induced cardiotoxicity combined with the prothrombotic properties of an immunomodulatory agent (lenalidomide) was observed to lead to significant thromboembolic events, requiring termination of this combination regimen (3). In patients affected by endometrial cancer, the combined use of a tyrosine kinase inhibitor (TKI), lenvatinib, and the ICI pembrolizumab raised concern for the long-term toxicity management (4). Therefore, an important goal of drug combining is, in addition to the increase in therapeutic efficacy, the limited increase in toxicity.

To this end, it would be desirable not to deviate too much from the approach, i.e. the study of the tumour microenvironment (TME), which led to the identification of immune checkpoints as targets of therapy. Starting from the study of the interactions between tumour cells and TME, it was possible to identify specific pro-tumour cellular circuits and molecules: surface proteins expressed, on the one hand, by neoplastic cells and, on the other, by immune response effector cells. Targeting these proteins by blocking antibodies (immune checkpoint inhibitors: ICIs) meant blocking an interaction limited to two specific cell types (although undesirable effects, such as autoimmune reactions, were then observed and were due to the existence of other cells, belonging to the immune system, which interact through the same checkpoints: PD-1/PD-L1, CTLA-4/B7, etc.). ICIs are therefore drugs with an appreciable selectivity for tumours. In some types of tumours, ICIs do not work satisfactorily and therefore synergistic treatments are sought. But the introduction of drugs, such as MEKI or TKI, whose targets belong to many intracellular pathways, leads to loss of selectivity for tumours and can indiscriminately target different normal cell types, thus producing high toxicity.

Instead, remaining anchored to the study of TME may allow the identification of other circuits and molecules, which are particularly relevant in tumours biology and much less for the homeostasis of most healthy cellular compartments. Drugs that target these circuits/molecules, alone or in combination with ICIs, can show a therapeutic potential, according to a design aimed at selectively targeting the tumour/TME interaction while preserving healthy cells and tissues of our body from damage.

## BAG3-targeting tools in combined therapies

The old and still current concept that tumours are wounds that do not heal (5) leads us to recognise the process that, together with the immunosuppressive features, characterizes TME: the inflammatory reaction (6, 7). Circuits and molecules that participate in the inflammatory process are highly relevant in TME. They play pro-tumour roles, through the production of some cytokines and growth factors, and through the deposition of collagen, which favours the tumour process in more ways than one: it stimulates the growth and the metabolism of neoplastic cells through the activation of the discoidin domain receptor 1 (DDR1)–NF-κB–p62–NRF2 signalling; creates excessive fibrosis, which in turn causes defective vasculature and hypoxia, resulting in the selection of apoptosis- resistant tumour clones; and hinders the penetration of anticancer effector cells (CTLs, etc.) (6–8). These circuits and molecules may therefore represent targets for acceptably selective anticancer therapies, which may affect healthy tissues only limitedly to inflammatory responses.

Among molecules that participate in inflammation, there are alarmins, or DAMPs (Damage- Associated Molecular Patterns), that include high mobility group proteins, heat shock proteins, various nucleotides/metabolites, and other factors. These are molecules that normally reside inside the cell, but are released to the extracellular space following cell death or, actively, upon cell exposure to a wide range of stressful stimuli. Outside the cell, alarmins play a different role than they do inside the cell: they trigger the activation of macrophages and other cells, leading to the release of cytokines, ROS and other mediators that foment inflammation (9). In growing tumours, neoplastic cells are exposed to metabolic, hypoxic, genetic and mechanical stresses. Therefore, DAMPs release is not unexpected.

**Figure 1:**
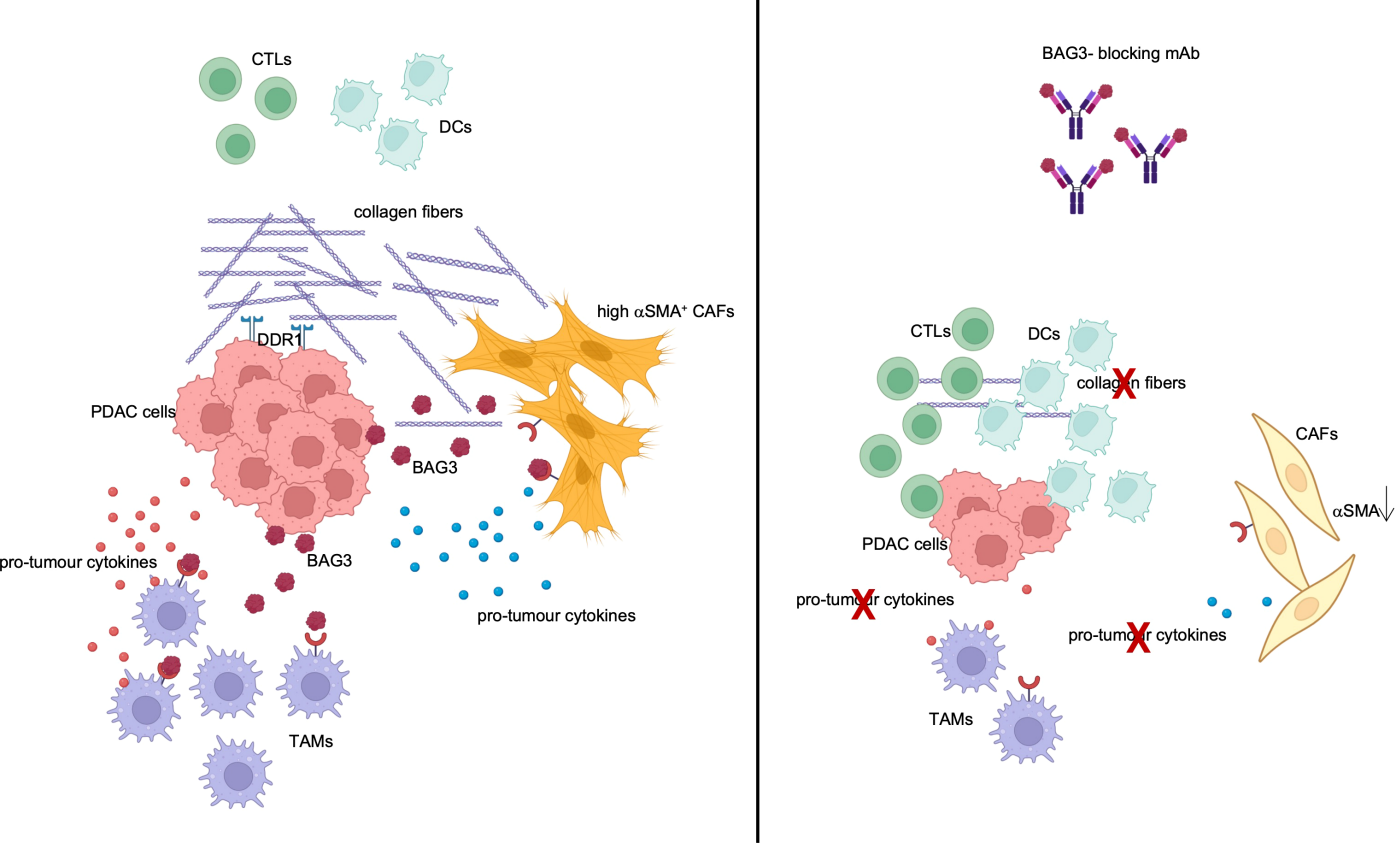
TME modulation by BAG3. BAG3 is released by PDAC cells and binds to its receptor on TAMs and CAFs, leading to their activation and to the release of pro-tumour cytokines and collagen. Created with BioRender.com.

Among alarmins, one came to our attention for the pro-tumour role shown in pancreatic adenocarcinoma (PDAC): BAG3 protein. This is expressed in PDAC cells, regulating cell survival, autophagy and other functions, and is also actively released (10). Interacting with its receptor (BAG3R) on tumour- associated macrophages (TAMs) and fibroblasts (CAFs), BAG3 stimulates the release of pro-tumour cytokines and collagen deposition (10–12). Administration of anti-BAG3 monoclonal antibodies (mAbs) to pancreatic cancer-bearing animals abated desmoplasia and induced infiltration of CD8^+^ T lymphocytes and dendritic cells into the tumour nest (10–13) (**Figure 1**). The effect on the decrease in tumour growth and metastatic spread was significant, even if partial. But, most importantly, no toxicity was observed with administration of anti-BAG3 mAb to healthy animals, since BAG3 is not secreted by healthy cells and is not found in healthy tissues (10, 13). This crucial feature candidates BAG3-targeted treatment as an ideal strategy for a combination therapy (14, 15). Indeed, experiments in several animal models showed that the combination of an anti-BAG3 mAb with an anti-PD-1 or anti-SIRPα mAb suppresses PDAC growth by more than 70% (11, 16).

## Conclusions

In our opinion, there is adequate evidence (10–16) that the BAG3/BAG3R axis is a pro-tumour circuit whose interruption harms the tumour process, but not the homeostasis of healthy cells in the body. This is therefore an example of how studying the TME-tumour interaction can highlight potential targets for non- or low- toxic therapeutic tools. These latter, alone or in combination with other drugs, can help address the multiple resistance mechanism present in primary and/or metastatic or recurrent tumours, preserving the patient from limiting toxicities.

## Author contributions

AR preparing the manuscript. LM choosing the journal. MM discussion of the manuscript. VL critical discussion of the reviewer’s comments. MD’A discussion of the manuscript. MT ideating the manuscript. All authors contributed to the article and approved the submitted version.

## References

[1] Wang Y Cui C Deng L Wang L Ren X. Cardiovascular toxicity profiles of immune checkpoint inhibitors with or without angiogenesis inhibitors: a real-world pharmacovigilance analysis based on the FAERS database from 2014 to 2022. Front Immunol (2023) 14:1127128. doi: 10.3389/fimmu.2023.1127128 37292205 PMC10244526

[2] Xie D Jiang B Wang S Wang Q Wu G. The mechanism and clinical application of DNA damage repair inhibitors combined with immune checkpoint inhibitors in the treatment of urologic cancer. Front Cell Dev Biol (2023) 11:1200466. doi: 10.3389/fcell.2023.1200466 37305685 PMC10248030

[3] Chan PP Sabus A Hemenway MS Chatfield KC White CJ Mirsky DM. Thromboembolic toxicity observed with concurrent trametinib and lenalidomide therapy. Pediatr Blood Cancer (2023) 70:e30190. doi: 10.1002/pbc.30190 36602034 PMC10519171

[4] Rimel BJ Crane EK Hou J Nakayama J MacDonald J Lutz K. Tyrosine kinase inhibitor toxicities: A society of gynecologic oncology review and recommendations. Gynecol Oncol (2023) 174:148–56. doi: 10.1016/j.ygyno.2023.05.007 37207499

[5] Dvorak HF. Tumors: wounds that do not heal: similarities between tumor stroma generation and wound healing. N Engl J Med (1986) 315:1650–9. doi: 10.1056/NEJM198612253152606 3537791

[6] Denk D Greten FR. Inflammation: the incubator of the tumor microenvironment. Trends Cancer (2022) 8:901–14. doi: 10.1016/j.trecan.2022.07.002 35907753

[7] Wen Y Zhu Y Zhang C Yang X Gao Y Li M. Chronic inflammation, cancer development and immunotherapy. Front Pharmacol (2022) 13:1040163. doi: 10.3389/fphar.2022.1040163 36313280 PMC9614255

[8] Su H Yang F Fu R Trinh B Sun N Liu J. Collagenolysis-dependent DDR1 signalling dictates pancreatic cancer outcome. Nature (2022) 610:366–72. doi: 10.1038/s41586-022-05169-z PMC9588640 36198801

[9] Murao A Aziz M Wang H Brenner M Wang P. Release mechanisms of major DAMPs. Apoptosis (2021) 26:152–62. doi: 10.1007/s10495-021-01663-3 PMC8016797 33713214

[10] Rosati A Basile A D'Auria R d'Avenia M De Marco M Falco A. BAG3 promotes pancreatic ductal adenocarcinoma growth by activating stromal macrophages. Nat Commun (2015) 6:8695. doi: 10.1038/ncomms9695 26522614 PMC4659838

[11] Iorio V Rosati A D'Auria R De Marco M Marzullo L Basile A. Combined effect of anti-BAG3 and anti-PD-1 treatment on macrophage infiltrate, CD8^+^ T cell number and tumour growth in pancreatic cancer. Gut (2018) 67:780–2. doi: 10.1136/gutjnl-2017-314225 PMC5868239 28801350

[12] Iorio V De Marco M Basile A Eletto D Capunzo M Remondelli P. CAF-derived IL6 and GM-CSF cooperate to induce M2-like TAMs-letter. Clin Cancer Res (2019) 25:892–3. doi: 10.1158/1078-0432.CCR-18-2455 30647085

[13] Basile A De Marco M Festa M Falco A Iorio V Guerriero L. Development of an anti-BAG3 humanized antibody for treatment of pancreatic cancer. Mol Oncol (2019) 13:1388–99. doi: 10.1002/1878-0261.12492 PMC6547619 30973679

[14] Gromisch C Qadan M Machado MA Liu K Colson Y Grinstaff MW. Pancreatic adenocarcinoma: unconventional approaches for an unconventional disease. Cancer Res (2020) 80:3179–92. doi: 10.1158/0008-5472.CAN-19-2731 PMC7755309 32220831

[15] De Marco M Turco MC Marzullo L. BAG3 in tumor resistance to therapy. Trends Cancer (2020) 6:985–8. doi: 10.1016/j.trecan.2020.07.001 32718905

[16] De Marco M Gauttier V Pengam S Mary C Ranieri B Basile A. Concerted BAG3 and SIRPα blockade impairs pancreatic tumor growth. Cell Death Discov (2022) 8:94. doi: 10.1038/s41420-022-00817-9 35241649 PMC8894496

